# The inframammary fold: Structure, clinical considerations, and reconstructive techniques

**DOI:** 10.1016/j.jpra.2025.11.022

**Published:** 2025-11-28

**Authors:** Camellia Richards, Sonam Patel, Hannah Markham, Jajini Varghese, Ramsey I Cutress

**Affiliations:** a Cancer Sciences, Faculty of Medicine, University of Southampton and University Hospital Southampton, Southampton, UK; b Histopathology Department, University Hospital Southampton, Southampton, UK; c The Breast Unit, Royal Free London NHS Trust, London, UK

**Keywords:** Inframammary fold, Anatomy, Reconstruction, Gender-affirming surgery, Double-bubble, Bottoming-out

## Abstract

The inframammary fold is a defining structure of breast position and form. Disruption, relocation and reconstruction of the Inframammary fold is therefore significant and may present surgical challenges. Furthermore, the inframammary fold’s anatomy, structure, relationship to other key thoracic landmarks and how these factors impact reconstruction is debated in the literature.

Several reconstructive techniques have been proposed since the IMF was first described as a distinct structure. These are commonly used in isolation or in combination, however there is a lack of direct comparison between different reconstruction methods. Much of the current literature is based on level IV or level V evidence often with small sample sizes.

The inframammary fold’s anatomy is discussed in this review on a macro- and microscopic level. Indications and procedures for inframammary fold reconstruction are discussed. The distinct challenges presented by different reconstructive techniques, gender-affirming surgery, inframammary fold insufficiencies and pathologies are considered.

## Introduction

The inframammary fold (IMF) is a primary anatomical landmark of the breast. It defines the shape, volume and curvature of the lower pole and influences the orientation and vertical position of the nipple–areola complex (NAC).^1^, ^2^, ^3^, ^4^, ^5^, ^6^

Anatomically, the IMF marks the junction between the inferior border of the breast and the anterior chest wall. As the boundary of the inferior pole, it is pivotal to breast form and must be preserved if possible or reconstructed when disrupted. Owing to its predictable relationships with the ribs, umbilicus and NAC, the IMF is also a key reference point in pre-operative planning.

Optimal aesthetic outcomes in augmentation and reconstruction depend on accurate IMF position, symmetry, and curvature. The fold routinely guides NAC repositioning, pocket design and implant selection. Despite its importance, descriptions of IMF location and the landmarks used to define it vary across the literature, and standard anatomy textbooks provide only cursory coverage or omit it altogether.^4^^,^^7^

## Methods

Relevant papers were identified using PubMed. The search term ‘inframammary fold’ was used in combination with key-words; ‘anatomy’, ‘reconstruction’, ‘gynecomastia’, ‘gender-reassignment’, ‘female-to-male’, ‘male-to-female’, ‘tuberous breast’, ‘double-bubble’ and ‘bottoming-out’.

The primary search was conducted in August 2023. Relevant primary studies of adult patients over 18 years of age were included. Studies found through citation in relevant papers were also included. Reports that were not published in English or where the full text was not available were excluded. The search was repeated in August 2025, seven further papers were included.

## Applied gross anatomy

### IMF position

For optimal aesthetic outcomes, the IMF should be symmetrical and proportional to key breast landmarks, the breast base, NAC and to the torso as a whole, particularly the sternum and umbilicus.^5^^,^^6^ Several anatomical references are used to describe and measure IMF position, including the pectoralis major, fifth to eight ribs, sternal notch (SN), NAC, and umbilicus. Oh et al.^8^ used supine CT and found the sixth rib most predictive of IMF position, with no variation by age or height. Wang et al.^9^, using magnetic resonance imaging (MRI), similarly supported rib-based prediction but located the IMF most often at the fifth intercostal space rather than the sixth rib.

Beyond ribs, the IMF has been referenced to bony and soft-tissue landmarks such as the SN, umbilicus, breast base, and NAC.^10^^,^^11^ Fujisawa^12^ proposed the TAP index, defined as the ratio SN-to-IMF/SN-to-umbilicus measured in standing and supine positions. In 145 patients, the mean TAP was 0.590, closely matching 0.575 derived from Westreich’s ‘aesthetically perfect breasts’ dataset.^13^ Standing measurements showed a lower IMF compared with supine, while body mass index (BMI) had no effect. Fujisawa suggested age-related descent of the IMF; this may reflect movement of soft-tissue reference points, such as the umbilicus, rather than true change relative to the ribs. The TAP index can aid IMF planning in augmentation and reconstruction, especially when both IMFs are disrupted.

Several other ratios have been published that can be used to inform localization of a new IMF and NAC position in reduction, augmentation and reconstruction. The ICE principle,^14^ Implant dimensions (I) − native breast capacity (C) = excess tissue requirement (E), aims to reproduce Mallucci’s 45:55 upper: lower pole ratio,^5^^,^^6^ guiding implant selection, pocket design and IMF incision placement so the scar rests within the new IMF. Although IMF relocation is ideally avoided, ICE provides a reproducible framework when it is necessary.

Mu’s ‘semicircle method’^15^ offers a rapid pre-operative marking technique for setting the contralateral new IMF during augmentation. After marking the midline and parasternal line (PS), the nipple-to-PS distance under maximal upward tension, to simulate implant stretch, is measured; the algorithm assumes the ideal lower pole is semicircular at nipple height, so nipple-to-PS equals nipple-to-new-IMF. When compared with Tebbetts,^16^ Randquist’s^17^ and ICE methods, it demonstrated consistency with Tebbetts, though not with Randquist or ICE, and yielded quick, easily applied markings with satisfactory aesthetic outcomes.

### IMF symmetry

IMF symmetry can be a key determinant of aesthetic outcome, particularly in breast augmentation. Interestingly, Yeslev^18^ found IMF level asymmetry in 95.4 % of pre-augmentation breasts, although the mean side-to-side difference was small, 4 mm.

Building on the interdependence between positions of the NAC and IMF, Li’s nipple–inframammary (NIMF) classification^19^ treats both landmarks as a unit and provides a surgical algorithm to standardize planning and optimize symmetry when using the transaxillary approach for breast augmentations (Table 1). The most common variation was NAC and IMF asymmetry in the same direction, for example, NAC and IMF positioned lower on the right side.^19^

**Table 1:** Nipple and Inframammary Fold classification for surgical planning in transaxillary augmentation mammaplasty.^19^

Inframammary fold type	Description of nipple and IMF symmetry
I	Asymmetrical nipple, asymmetrical IMF in the same direction
II	Symmetrical nipple, asymmetrical IMF
III	Asymmetrical nipple, symmetrical IMF
IV	Asymmetrical nipple, unapparent IMF

While many IMF height differences are minor and clinically imperceptible, visible discrepancies should be documented. A shared plan for correction should be discussed with the patient and is essential for aligning expectations and improving satisfaction.^20^, ^21^, ^22^ If asymmetries are not discussed, especially in elective cosmetic cases, patients may notice them post-operatively and misattribute pre-existing differences to the augmentation, leading to dissatisfaction.^22^ Pre-operative assessment should document base diameter, IMF level, NAC position and volume asymmetries, and incorporate targeted corrections: implant selection, pocket design and controlled IMF modification. This approach enables realistic counselling about the degree of achievable symmetry and supports consistent surgical decision-making.

## Microscopic anatomy

### The histological basis of the IMF

Since the IMF was first described in 1845 by Sir Astley Cooper, stating that at ‘the abdominal margin, the gland is turned upon itself at its edge, and forms a kind of hem’, there has been debate over the IMF’s true anatomical and histological structure.^23^

Early cadaveric work proposed a discrete ligament. Maillard and Garey^24^ described a crescentic band between the dermis and the anterior surface of pectoralis major, while Bayati and Seckel^25^ reported an inframammary crease ligament arising medially from the periosteum of the fifth rib and laterally from fascia between the fifth and sixth ribs.

Subsequent studies did not confirm a constant, isolated ligament in the IMF region.^1^, ^2^, ^3^ Instead, they support Lockwood’s concept^26^ of zones of superficial fascial adherence as the functional unit that generates the fold. Nava^1^ described a deepening of the superficial fascia forming a pseudo-ligamentous band within the breast’s anterior capsule, the fascia mammae, arguing this is not a true ligament. Boutros^27^ identified a dermal collagen condensation that increases in density toward the pectoralis fascia. Muntan^2^ reappraised the histology and depicted the IMF as a fusion of superficial and deep fascia with the dermis, with collagen fibers from the superficial fascia inserting into the dermis at or just below the IMF. Interestingly, no organized IMF connective structure was seen in male specimens.

**Figure 1:**
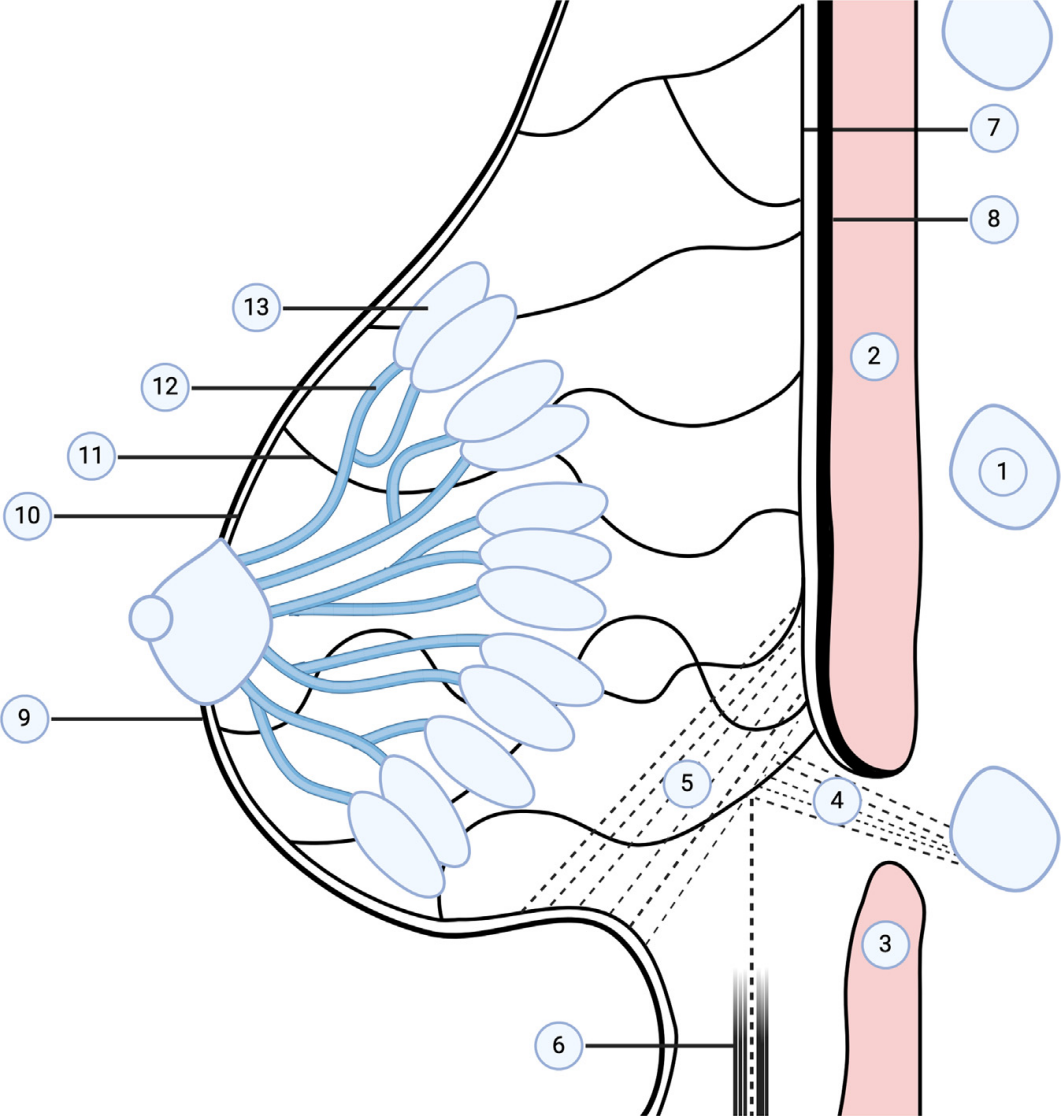
Schematic representation of putative fascial structures of the inframammary fold (IMF) and inferior pole of the breast based on studies described by Takaya(3) and Salgarello(27). It is proposed that double-bubble appearance can occur in submuscular implant placement when the inferior wing (4) is disrupted but the superior wing largely intact. Bottoming-out can occur in subglandular implant placement with disruption of the superior wing (5) and inferior prolapse of the implant and in sub-glandular implant placement with disruption of bot superior and inferior wings (4 and 5). Created with BioRender.com. Key of structures: 1. Ribs, 2. Pectoralis major, 3. Rectus femoris, 4. Fascial condensation: Inferior wing, 5. Fascial condensation: Superior wing, 6. Superficial fascia (Scarpa’s fascia), 7. Deep layer of superficial fascia, 8. Deep fascia, 9. Skin: epidermis, dermis, subcutaneous fat, 10. Superficial fascia: Camper’s fascia, Scarpa’s fascia, 11. Coopers ligament, 12. Lactiferous ducts, 13. Glandular lobules.

More recently, Takaya^3^ combined radiological, gross and microscopic analysis to describe the IMF as a multilayered ‘hanging’ construct of finely branched fascial septa separating fat lobules in the IMF region. Posteriorly and cranially, these septa coalesce and blend with pectoralis major near the fourth-to-fifth ribs, limiting skin mobility and helping maintain breast shape. Small, specialized subdermal fat lobules were present beneath the IMF in every sagittal section, more extensive laterally, toward the anterior axillary line, and minimal medially near the sternum. Takaya proposed that variation in lobule size and septal fusion together sculpt the characteristic IMF contour (Figure 1). Rather than a single ligament, contemporary evidence supports the IMF as a fascial condensation extending from dermis to superficial fascia, superior wing, and to the rib periosteum, inferior wing (Figure 1).^3^^,^^28^^,^^29^

### Breast tissue within the IMF and oncological relevance

Whether breast parenchyma extends into the IMF, and the implications for mastectomy remains debated.^2^^,^^30^, ^31^, ^32^, ^33^, ^34^, ^35^ Establishing presence and volume of any glandular tissue at the IMF is clinically important. In a 12-cadaver series, Muntan found no breast tissue within the IMF.^2^ In contrast, Gui^31^ reported breast parenchyma in 11/50 IMF mastectomy specimens, and Carlson identified parenchyma in 13/24 samples; however, its mean volume was 0.04 % of total breast volume, suggesting a minimal, anatomical variant.^32^

Using MRI in 290 pre-mastectomy breasts, Dassoulas observed that the parenchyma terminated above the IMF in 82.7 % and at the IMF in 17.3 %.^35^ Few studies isolate IMF-specific samples. Joo’s review attributed 2.9 % of recurrences to the lower breast/IMF region without IMF-only data,^33^ Watson reported 1.7 % involving the IMF,^34^ and Behranwala recorded 0.7 % primary IMF tumors with no recurrences at 3-year follow-up.^30^ None correlated histological subtype with IMF involvement.

Given the small, inconsistent prevalence and minimal volume of IMF parenchyma and low reported recurrence, less than 3 % of overall recurrence, standard mastectomy techniques appear adequate for removing clinically significant tissue at the IMF.^33^ Definitive IMF-specific recurrence rates remain uncertain. Larger, IMF-targeted studies are warranted, including analyses of recurrence location, and prevalence studies in non-cancer cohorts. Potential donation cohorts include specimens from female-to-male chest masculinization surgery.

## The IMF: clinical implications for breast surgery

The IMF is a key supportive element of the breast footprint and a principal determinant of lower-pole shape and contour. In most operations the IMF should be identified and preserved. If the fascial condensation is intentionally or inadvertently breached, reconstruction is often required to restore form (Appendix 1). Below we outline considerations across resectional, ablative and reconstructive settings.

### Mastectomy

There are several types of mastectomy including total (simple), skin-sparing and nipple sparing. Skin/nipple sparing approaches usually preserve the native IMF and skin envelope to facilitate reconstruction. Inadvertent violation may necessitate IMF reconstruction. In contrast a total, skin sacrificing, mastectomy has a greater risk of IMF disruption. Deliberate effacement may be appropriate to create a flat contour.^36^ When reconstruction is planned, the degree of IMF dissection must be balanced with the risk of IMF disruption. The longevity and aesthetic outcome of breast reconstruction depend on an intact IMF.

### Gynecomastia

Techniques adapted from female breast reduction including periareolar mastopexy, subcutaneous mastectomy and inferior pedicles are commonly used to treat grade II-III gynaecomastia.^37^, ^38^, ^39^, ^40^, ^41^ The IMF is ideally conserved with these techniques and incisions may be hidden within the IMF contour. Where there is a distinct IMF crease, or skin excess, IMF disruption may occur with excision.^37^^,^^40^ Muntan’s male cadaveric work described collagen fibers from superficial fascia inserting into the dermis along the IMF,^2^ echoing Lockwood’s adherence-zone concept.^26^ Despite the clinical prominence of the fold in gynecomastia, histological comparisons between normal and gynecomastoid male IMF remain scarce.^37^, ^38^, ^39^, ^40^

### The IMF in female-to-male gender-affirming surgery

In female-to-male chest surgery, similar to total mastectomy, elimination of the IMF is necessary to achieve the desired male chest contour. This is performed via bilateral mastectomy where the large volume of skin and tissue removed allows the IMF to be completely resected. Incision scars are usually placed to highlight the contour of pectoral muscles creating a masculine chest wall appearance.^42^

### Cosmetic augmentation

In cosmetic augmentation procedures IMF position is assessed and if acceptable special care is taken to prevent disruption. Where possible the original IMF should be preserved especially in cosmetic augmentation surgery. Placement of the surgical incision just above the IMF can facilitates IMF preservation, and allows the scar to lie on the undersurface of the breast where it will be less visible and minimize irritation from underclothing or bra underwiring.^43^ Even when planned, IMF disruption introduces risks of insufficiency deformities including double-bubble and bottoming-out.

In certain cases, IMF disruption cannot be avoided; in order to correct conditions such as unilateral hypomastia or a constricted lower pole. At times it may need reinforcement to carry the additional weight of an implant. In some circumstances IMF position will need to be intentionally lowered to recruit skin into the lower pole to accommodate a larger implant than the dimensions of the natural breast allow, or to symmetrize with the contralateral IMF.

Phillips^44^ describes an IMF classification method for breast augmentation, based on clinical appearance, and in relation to the risk of double-bubble deformity (Table 2). This classification system is used by Phillips to aid surgical planning, implant selection, and IMF relocation.

**Table 2:** Phillips’ inframammary fold classification system.^44^

Inframammary fold type	Visible inframammary fold in the standing position	Effacement of IMF with arm elevation
Type 0 (F0)	No	N/A
Type 1 (F1)	Yes	Complete
Type 2 (F2)	Yes	Incomplete
Type 3a (F3a)	Yes	No
Type 3b (F3b)	Yes	An IMF that extends superolaterally resulting in a fixed lateral border that remains with arm elevation

Where the IMF is poorly defined (F0-F1) it can be relocated more easily with less disruption and hence lower risk of double-bubble deformity. In larger breasts with a well-formed IMF, F2-F3, the native IMF is ideally conserved as these patients are high risk for lower pole complications. However,^28^^,^^45^ where IMF lowering cannot be avoided, minimal lowering and a conservative approach to implant volume and projection is preferable. Deep dissection inferior to the original IMF and incorrect implant placement can lead to a double-bubble appearance.^45^

Fixating and repositioning the IMF can be challenging, particularly due to its dynamic nature and instability during scar maturation. During this period supportive aids such as underwired bras have been beneficial for stabilizing the new fold, preventing excess tension leading to IMF lowering and ensuring that scar formation occurs at the correct level.

Interestingly, in a mastopexy procedure where the horizontal scar limb is placed in the IMF, a combination of upward traction due to skin removal and reduced tension on the IMF due to tissue removal may often lead to the appearance of a raised IMF.^46^ The IMF is not routinely breached in these surgeries therefore its anatomical position and integrity often remains the same.

### Tuberous breast

Tuberous breast is a rare and reconstructively challenging congenital breast abnormality that presents at puberty. A fibrous constricting ring at the breast base prevents normal horizonal and vertical breast growth.^47^, ^48^, ^49^ This results in characteristics including tubular shape, breast parenchyma hypoplasia, asymmetry, skin deficiency vertically and horizontally, NAC protrusion, a constricted breast base and elevated IMF. Patients can present with varying degrees of severity and combinations of these anomalies. However, reducing constriction, its defining characteristic, and deciding on new IMF level placement can be some of the most challenging issues.

Von Heimburg’s classification is routinely used, categorizing severity based on the mammary base and quadrant volume.^47^ Surgical planning is often complex commonly involving multiple reconstructive techniques to reduce the size of the NAC, release and lower the constricted IMF and correct breast volume deficiency (Appendix 1).

As described in Grella’s review^50^ surgical techniques have been proposed often utilizing periareolar mastopexy with breast implant to correct NAC protrusion and breast ptosis. The constricted breast base is often released with transection or original IMF scoring, and dissected down until the new IMF level is reached.^48^^,^^50^, ^51^, ^52^

This patient cohort has increased risk of double-bubble deformity as the imprint of the original constricted IMF base is hard to eliminate. Fat grafting has frequently been used to treat double-bubble and provide additional volume in areas of hypomastia or significant asymmetry in patients with or without tuberous breast.^53^

This patient cohort presents a variety of IMF reconstructive challenges. Notably, the constricted lower pole and raised IMF must be corrected in order to achieve a natural breast shape and position. If these issues are not adequately accounted for, other aspects of reconstruction including use of implants or fat grafting will not achieve a satisfactory result.^37^^,^^40^^,^^54^

### The IMF in male-to-female gender-affirming surgery

There are several differences to consider when planning breast augmentation in transgender women compared to cis-women. Similar to IMF level adjustment in cis-women, Coon^55^, Kanjoor^56^ and Jagasia^57^ describes obliteration of the original IMF with scoring. With Coon’s method the new IMF level was defined with anchoring sutures to the rib periosteum. This study describes lower pole constriction and risk of double-bubble when the original IMF is not sufficiently dissected and implant displacement when the IMF is not adequately reconstructed. It was noted that if the IMF was not lowered it resulted in increased upper pole volume and short nipple-to-IMF distance leading to NAC inferiolateral orientation.

Similar to Coon, Kanjoor describes scoring the original IMF where the fold was tight and inflexible. In 175 of Kanjoor’s 177 patients an inframammary approach was used for pocket dissection.^56^ The inframammary incision was closed in three layers: fascial approximation and subdermal approximation using continuous sutures followed by subcuticular sutures. Unlike Coon, periosteal sutures were not used to define the new IMF, despite this there were no reports of bottoming-out deformities. This could be due to limited patient follow-up. However, Jagasia’s 140 patient study also did not use sutures to define the new IMF.^57^ In Jagasia’s twelve-month minimum follow-up period bottoming-out was not among the reported complications. Use of the subfascial plane as the preferred dissection plane may explain this. Jagasia theorized that the thickened pectoralis fascia in patients assigned male at birth compared to cis-women, provided a strong support to hold the implant at the correct level, preventing implant migration.

It is important to highlight surgical challenges from breast development secondary to estrogen therapy. This breast development was felt to be comparable to the gynecomastoid breast potentially causing an ‘unnatural double mound’ formed from breast parenchyma lying anterior to an implant with visible implant edges due to the absence of breast tissue at the IMF and breast peripheries.^55^ Coon suggested that aggressive scoring of the breast tissue, fat grafting and dual-plane submuscular placement helped reduce these issues, with Jagasia recommending at least 1 year of hormonal therapy to achieve sufficient glandular tissue development prior to augmentation.^57^

## Insufficiencies of the IMF

### Double-bubble

Double-bubble is an uncommon complication of implant-based breast reconstruction and augmentation. This deformity is seen as an abnormal crease running across the lower pole, creating the appearance of a second IMF.^2^ The double convexity at the lower breast pole is caused by the indentation from the original IMF creating a ‘waist’ over the implant. People with particular breast morphologies including tuberous breast or constricted base width are predisposed to the double-bubble phenomenon. Over dissection of deeper IMF connections pre-disposing to implant prolapse inferiorly, especially along with incomplete release of the superficial attachment of the IMF to the dermis followed by concomitant descent of the implant posterior and inferior to this attachment, create this appearance.

### Preventing double-bubble

Swanson^43^ describes a supra-IMF incision, instead of the tradition IMF incision, in primary subpectoral breast augmentation to avoid disrupting IMF fascial connections, preventing double-bubble. The study showed low short-term risk, 0.6 %, of double-bubble, compared to studies which disrupted and then repaired the IMF.^58^ The plane the implant pocket lies in is another key consideration for the risk of double-bubble deformity. Salgarello states that a subglandular pocket greatly reduces risk of double-bubble, whereas the risk is increased when the implant pocket is dissected in the submuscular or dual plane.^28^ Although disrupting the IMF should be avoided if possible, augmentation and reconstruction may involve lowering the IMF to recruit skin. In these cases, care should be made to perform full IMF dissection; both superficial fibers to the dermis and deeper fascial connections to the chest wall, prior to reconstruction.^45^

### Bottoming-out

Bottoming-out is another similar, but distinct, inferior implant malposition deformity. The breast implant becomes inferiorly displaced below the original IMF level; this results in disproportionate inferior breast pole fullness and an increase in the nipple-to-new-IMF distance giving the appearance of excessively high nipples.

Two factors contribute to bottoming-out, excessive pressure on the fold and a weak IMF. Large implants or a pocket that is too small for the implant can lead to increased pressure on the fold. Various factors can reduce IMF strength. If the IMF is relocated, often to accommodate a larger implant or if mastectomy does not leave adequate tissue for reconstruction, inadequate reconstruction can lead to insufficient implant support. In particular disruption to the superficial attachments to the dermis and the deeper attachment to the chest wall muscle allow the implant to descend.^28^ Other factors including natural ptosis, pregnancy, smoking and previous breast augmentations also cause IMF thinning and tissue weakness increasing the risk of IMF failure and implant drop.

Salgarello^28^ highlights the risk of superior wing disruption (Figure 1) and therefore bottoming-out when dissecting the subglandular pocket. To reduce this risk, they suggest dissecting the plane slightly more superficially at the IMF level when lowering the fold. Salgarello also suggested beginning dual-plane pocket dissection above the subpectoral fascia in the suprafascial plane and entering the subpectoral plane 1 centimeter above pectoralis major’s inferior margin, preserving the inferior and superior wings, avoiding bottoming-out and double-bubble.

## IMF reconstruction techniques

Several methods for IMF reconstruction have been proposed. These techniques can be broadly categorized into three distinct reconstruction methodologies: local tissue rearrangement,^58^, ^59^, ^60^, ^61^, ^62^, ^63^ suture suspension,^64^, ^65^, ^66^, ^67^, ^68^, ^69^, ^70^, ^71^ and use of an acellular dermal matrix^72^, ^73^, ^74^, ^75^, ^76^, ^77^ (ADM) (Appendix 1). External devices have been classified separately in other reviews^4^ however the majority of papers on external device use are case reports and level V evidence which we have not included in this review other than to mention here. Singular or a combination of these techniques may be used to reconstruct the IMF. The method chosen is influenced by several factors including type of prior mastectomy, simple or skin-sparing, use of an implant and surgeon preference. Further details and summary of local tissue rearrangement, suture techniques and ADM used in IMF reconstruction can be found in Appendix 1; in particular focusing on interrupted and running anchoring techniques and ‘drawstring’ suture methods (Table 3).

**Table 3:** A table summarizing studies that describe suture techniques and methods used for inframammary fold reconstruction as part of breast reconstruction and/or breast augmentation.

Author	Level of evidence	Patient number	Procedure	Suture technique	Suture material	IMF reconstruction method
Pennisi^59^^,^^a^	5	-	Post mastectomy reconstruction	Anchoring (interrupted)	Non-barbed	De-epithelialized margin of inferior flap sutured to the pectoral fascia.
Ryan^60^^,^^a^	5	63	Post mastectomy reconstruction	Anchoring (interrupted)	Non-barbed absorbable	De-epithelialized dermis from the inferior flap directly sutured to periosteum at the level of the new IMF.
Versaci^63^^,^^a^	5	2	Post mastectomy reconstruction	Anchoring (interrupted)	Non-barbed	Internal method: interrupted sutures through the subcutaneous fat, dermis, and periosteum at the level of the new IMF, or Ryan’s method.
Handel^61^^,^^a^	5	2	Post mastectomy reconstruction	Anchoring (interrupted)	Non-barbed non-absorbable	An inferior flap is dissected in the deep plane anterior to the thoracoabdominal fascia, 2–3 cm above the desired IMF location. A flap including the deep subcutaneous tissues and superficial fascia is created extending to just proximal to the desired IMF level. This flap is elevated and sutured to the chest wall with interrupted figure of eight sutures.
Nava^1^^,^^a^	4	100	Post mastectomy reconstruction	Anchoring (running)	Non-barbed absorbable	Capsulotomy scoring internally until the hypodermic level of the inframammary crease is dissected off the superficial fascial plane. A suture is run medially to laterally along the new IMF. Two sutures both starting in the midclavicular line on the IMF run in opposite directions, one medially and one laterally along the new IMF. These sutures fix the superficial fascia to the thoracic wall.
Hirsch^20^^,^^a^	4	45	Post mastectomy reconstruction	Drawstring	Barbed Absorbable	Sutures begins in the middle of the IMF and run in opposite directions, dermis sutured to deep tissue, rectus fascia/periosteum. Tension is adjusted, when adjustment is complete the suture is briefly run backwards to lock it.
Terao^64^^,^^a^	4	102	Post mastectomy reconstruction	Drawstring	Barbed Non-absorbable	Sutures fixated to deep tissues medially, multiple passes are made through the dermis laterally then adjusted—no doubling back, unidirectional.
Sarfati^78^	5	-	Post mastectomy reconstruction	Anchoring (running)	Non-barbed absorbable	Hammock technique: a single suture run along the curve of the planned new IMF. The suture is fixated to the chest wall at the lateral and medial IMF edges. Further anchoring stitches between the subcutaneous fascia and chest wall can be added to adjust if needed.
Hamdi^65^^,^^a^	4	180	Post mastectomy reconstruction	Drawstring/purse-string	Non-barbed absorbable	Suture is fixated laterally in axilla region, first pass (superficial plane) – the suture is passed through cannula subcutaneously, lateral to medial along the new IMF curve passing under deep dermis. At the superior pole of the breast the suture is passed from the superior pole (at the medial IMF) to the initial insertion point in the axilla. The suture will lie superior to pectoralis major. The second pass (deep plane)—the suture is then passed along the IMF in a deep plane just superficial to the fascial layers, at the superior breast pole the suture is passed deep to pectoralis major. The suture is then tightened, tied and the ends buried in the deep tissues.
Ismagilov^79^^,^^a^	4	321	Post mastectomy reconstruction	Anchoring (interrupted)	Non-barbed non-absorbable	Post use of tissue expander (TE). The anterior/ventral surface of the posterior sheet of the TE capsule was dissected, the posterior/dorsal surface of the posterior capsule was dissected away from the chest wall. The posterior capsule was pulled up and fixated at a higher position to the chest wall with interrupted sutures.
Nakajima^69^^,^^a^	4	9	Post mastectomy reconstruction	Anchoring (interrupted)	Non-barbed absorbable	Each suture is fixated, no set starting point. Two incision points are made 1 cm apart, A and B. The suture is passed through the chest wall (ideally periosteum or perichondrium) via point A, suture exist via point B, then the suture is inverted and passed through the dermis and superficial layer of the subcutaneous tissue, exiting at point A. The suture is tied and buried in the subcutaneous tissue. This is repeated along the IMF to define the IMF. Four to five sutures used along the medial and central parts of the IMF and one to two sutures along the lateral IMF.
Visconti^67^^,^^a^	3	51	Primary augmentation	Drawstring	Barbed Absorbable	One suture is looped and fixated medially to pectoralis major, one suture is looped and fixated laterally to serratus anterior, the four ends of the two sutures are run percutaneously towards the midmammary line, the first free ends of the medial and lateral suture cross in the midmammary line and then exit, whereas the other medial and lateral threads exit near the midmammary line before crossing, the threads are then drawn to tighten, and cut at skin level.
Tomita^66^^,^^a^	4	20	Post mastectomy reconstruction	Drawstring	Barbed Non-absorbable	The suture is fixed at the medial end of the IMF to costal cartilage. The suture is passed medially to laterally through the subcutaneous fat. Where the IMF is shallow the suture is passed through the deep layer of subcutaneous fat and the superficial layer where IMF is deep. The suture exits laterally, is tightened, and buried under the skin.
Qin^68^^,^^a^	3	184	Primary augmentation	Anchoring (running)	Non-barbed absorbable	A four-layer closer technique as opposed to the traditional three-layer technique. A continuous suture is used to suture Scarpa’s fascia to the deep fascia (superficial pectoralis fascia) to secure the IMF. Interrupted sutures are used to close Camper’s fascia and then the deep dermis. Running intradermal sutures are used to close the epidermis.
Pinto^71^^,^^a^	4	88	Post mastectomy reconstruction, contralateral breast augmentation tuberous breast	Anchoring (interrupted)	Non-barbed non-absorbable	Several sutures anchoring the dermis to chest wall used with liposuction to define the IMF.
Hudson^80^^,^^a^	4	56	Post mastectomy reconstruction and breast reduction	Anchoring (interrupted)	Non-barbed absorbable	The IMF is reinforced with four to five sutures anchoring the subdermal layer and superficial fascial system to the pectoral fascia and periosteum. Sutures are placed along the line of the new IMF; at the breast meridian, the mid-point of the medial half of the IMF and along the lateral IMF.
Ospital^81^^,^^a^	5	-	Post mastectomy reconstruction	Anchoring (interrupted)	Non-barbed non-absorbable	Access to the IMF is gained through an incision over the old mastectomy scar. Six to eight deep Vicryl 1 interrupted sutures are passed through the full thickness of the thoracoabdominal flap at a level below the intended IMF then attached to the periosteum at the desired new IMF level. The second row of interrupted Vicryl 1 sutures are passed superficially. Six to eight sutures are passed through the dermis of the flap and anchored to the abdominal wall at the level of the new IMF.
^62^	4	213	Augmentation mastopexy	Anchoring (running)	Barbed	A barbed suture is used to attach the breast pillars to Scarpa’s fascia and the aponeurosis of pectoralis major to form the new IMF. The technique advises different methods to eradicate the old IMF and stabilize the tissue below the new IMF depending on length between old and new IMF. If the IMF is raised a small amount, less than 2 cm, the entire old IMF is released and sutured to the abdominal wall, if 2 to 4 cm ascent is needed and no breast tissue is present below the new IMF, liposuction is performed before suturing to the chest wall. If breast tissue is present this is resected in addition to old IMF release. Adhesional stitches are used to prevent dead space. If the new IMF is greater than 4 cm higher than the old IMF; liposuction, glandular tissue resection, old IMF release and adhesional stitches are all used to anchor the tissue to the abdominal wall.
Urbain^70^^,^^a^	3	26	Post mastectomy reconstruction	Anchoring (running)	Non-barbed absorbable	Liposuction in the subdermal plane of the subcostal area is performed to improve IMF definition before fixation of the new IMF. Percutaneous fixation along the new IMF is performed using a 1.0 triangular needle PDS loop. The suture is passed multiple times along the length of the new IMF, lateral to medial, and then medial to lateral, anchoring the dermis to pectoralis major or periosteum. The previous needle exit point is used as the next suture pass’s entry point, therefore the suture is not externally exposed.

a Indicates that the publication contains a schematic and/or diagram of the technique.

## Conclusion

Recent histological and gross anatomical studies generally agree that the IMF should not be considered a true ligament and rather a network of dense fascial condensation from the dermis and superficial fascia to the chest wall.^1^, ^2^, ^3^^,^^28^ The IMF plays a crucial role in reconstructive and aesthetic breast surgery, contributing to the shape, fullness, and lower pole definition, as well as the positioning of the nipple. This review provides an overview of IMF anatomy, current surgical techniques, and the challenges involved in IMF reconstruction emphasizing that where possible the IMF should be preserved.

A significant challenge from the literature is the small sample sizes of studies evaluating these techniques, as well as the lack of long-term outcome data. Further research is needed to assess the risks of malposition deformities associated with different incision approaches and dissection techniques, particularly in patients with conditions like tuberous breasts. This review also discusses strategies to minimize deformities such as bottoming-out and double-bubble deformity. Further investigation would greatly improve the understanding of these complex surgical decisions and improve outcomes for patients undergoing IMF reconstruction.

When the IMF is disrupted or a new IMF level is planned the IMF must be reconstructed to achieve the best aesthetic outcomes. A thorough preoperative assessment of IMF configuration is essential, including identifying any pre-existing asymmetry, to determine whether, and which corrective intervention may be required during surgery. IMF creation can also be combined with adjunctive procedures; such as an ADM in resectional surgery to anchor an implant, or dermal liposuction, to enhance IMF definition (Appendix 1). Understanding various techniques for determining the ideal position of a new IMF, based on anatomical landmarks and the contralateral breast, can be particularly helpful when addressing complex cases.

## Declaration of competing interest

None declared.

## Appendix Supplementary materials

Image, application 1
