# Supplementary material for: The inframammary fold: Structure, clinical considerations, and reconstructive techniques
Source: JPRAS Open. 2025 Nov 28;48:457–72. doi: 10.1016/j.jpra.2025.11.022 (PMC12803885; doi:10.1016/j.jpra.2025.11.022)
Supplement: Supplementary file 1 [file mmc1.docx]

**The inframammary fold: Structure, clinical considerations, and reconstructive techniques**

**Richards C. et al**

**Appendix 1: Inframammary fold reconstruction techniques**

**Local tissue rearrangement**

The earliest description of intramammary fold (IMF) reconstruction involves local tissue rearrangement and suturing of tissue to the chest wall at the new desired position. Pennisi^59^ described de-epithelializing the skin along the desired IMF position, turning it upward, and suturing it to the chest wall. The skin edges are then approximated to form the reconstructed IMF.

Ryan^60^ described a similar approach which involved undermining the chest and upper abdominal tissue and advancing the de-epithelised lower thoracic flap. This was then secured at the new-IMF and anchored to the chest wall. Assessment of long-term outcomes using 3D imaging showed improved and stable outcomes. However, the scars, positioned where the new-IMF sits, run the length of the new-IMF and can lead to chronic irritation. There is also the additional consideration of possible devascularisation of the lower mastectomy flap when there is an additional anterior scar.

Internal approaches, describe the advancement of tissue, without the creation of an external scar at the new-IMF position.  Handel and Jensen^61^ described creation of an internal lipofacial flap incorporating the Scarpa’s fascia. This flap is advanced and secured to the chest wall to create a new-IMF at the desired location. Similarly, Nava’s technique^1^ involves capsulotomy and superficial fasciotomy to create a fascial flap at the desired IMF position which is elevated and secured to create the new-IMF. There are several other techniques described with minor variations which are detailed in Table 2.

Bark’s retrospective study^62^ presents a technique to aid augmentation mastopexy in patients with a low-lying IMF, below the sixth rib. As with previous local tissue rearrangement techniques^60,61^, tissue deep to Scarpa’s fascia, between the new-IMF level and the original fold, is resected. This thinned tissue is fixated to the abdominal wall. The study advises different combinations of IMF release, glandular tissue dissection, adhesional stiches and liposuction depending on the distance between old and new-IMF. Barbed sutures are used to attach the new-IMF to Scarpa’s fascia and pectoralis major (Table 2).

Versaci^63^, combined both external and internal approaches when reconstructing larger breasts. The posterior capsule is incised and secured at the desired position as described above. This is combined with the external approach of de-epithelisation and advancement of the inferior thoraco-abdominal flap which is then anchored to the rib periosteum. The superior mastectomy flap is sutured to the edge of the deepithelialised skin.

Local tissue rearrangement can create a smooth, well-defined IMF, recruiting more tissue into the lower breast pole and enabling implant coverage.

**Suture Suspension**

Suture suspension, through an internal or external approach is commonly used in IMF reconstruction^64-71^. Although there is variation in suture methods the foundational principle involves placing singular or multiple sutures in the superficial tissues along the new-IMF to define the new fold.

The ‘drawstring’ technique is a commonly described method^64-67^. Modifications and additions to the drawstring technique have been proposed by several authors since Terao^64^, as detailed in Table 2. The characteristic feature of a drawstring technique is the tightening of the suture after placement to create the desired IMF definition. This technique may involve fixation to muscle^65-67^ or periosteum^64^. Risk of pneumothorax was highlighted with anchoring to the chest wall^61^ but not in comparison to other anchoring methods. Other drawstring methods did not comment on the choice of muscular^65,67^ over periosteum anchoring^64^.

Fixation of the suture at the medial or lateral end of the IMF varied. Tomita^65^ suggested that medial fixation allowed greater breast mobility, which was particularly important when using non-absorbable sutures, to prevent a tethering sensation and discomfort at the fixation point.

Different suture materials have been used for the drawstring technique. Barbed sutures^64,65,67^ eliminate the need for knot tying and distal suture fixation to deep structures, as the barbed suture adheres to its surrounding tissue along its length^16^. However, the risk of post-operative suture slackness due to prolonged and excessive tension on the barbed suture was noted with this material^64,65^.

Hamdi^65^ and Visconti^67^ used an absorbable barbed suture, relying on scar tissue forming in the indentation of the absorbed suture to maintain long term IMF integrity. Dimpling of skin due to the suture tightness was observed in the short term but disappeared with suture absorption^66,67^. However, Tomita’s drawstring technique favoured non-absorbable sutures reporting reversion of the IMF to its original position after suture absorption with absorbable sutures^66^. Although barbed and non-absorbable sutures provide more secure anchoring, they are associated with a greater inflammatory response^68^.

The number of sutures used and suture exit position along the IMF varies in different drawstring methods. Visconti^67^ used two sutures exiting in the central IMF region to reduced tension on the individual suture lines, reducing the risk of suture slackness and scalloping, particularly in the short term.

The variations of the drawstring method aim to balance the need for good IMF integrity and definition with the risk of suture slackness, skin dimpling and scalloping caused by excess tension on sutures. The main advantage to the drawstring technique is that it is a scarless technique which is particularly beneficial in patients where the IMF is visible due to smaller breast size^64^.

Another suture technique is the use of percutaneous anchoring sutures^69,71^ to fixate the subcutaneous tissue to deep structures along the IMF as shown in Table 2. With Nakajima’s^69^ and Urbain’s^70^ methods, anchoring sutures are placed percutaneously along the IMF forming a new scarless IMF. As with the drawstring technique, scalloping is highlighted as a complication from sutures under excessive tension^69^. This was usually a short-term complication that gradually disappeared as suture tension reduces. A key benefit of Nakajima’s^69^ percutaneous method is that the suture technique can be used after implant insertion, although it does carry a risk of implant perforation.

Ospital’s method^73^ is another technique which expands on Nava’s, presenting a double fixation technique using a thoracic adipofascial flap. Two rows of sutures are used, deep and superficial. The deep sutures secure the new-IMF to the periosteum. This allows the second superficial row of sutures, anchoring the dermis to the abdominal wall, to define the new-IMF without tension, unlike Nakajima’s method.

Adjuncts to suture methods, particularly fat grafting, have been used to enhance IMF definition^66,67,62^. Pinto^71^ described the use of dermal liposuction along the planned IMF curve to define the new fold. They found that this technique recreated the anatomical characteristics of the natural IMF resulting in long-lasting satisfactory aesthetic outcomes. In some cases of severe asymmetry this was used alongside sutures anchoring the dermis to the chest wall at several points along the new IMF to further distinguish the curve.

Suture methods are used both in isolation and in addition to other methods of IMF reinforcement and re-creation^64-69^. Although, different suture methods have described their individual advantages and limitations no clinical studies comparing the efficacy of different techniques were found. However, the key value of these techniques is that they use closed approaches avoiding IMF scars and consequently cosmetic and symptomatic sequelae compared to tissue arrangement techniques.

**Acellular dermal matrix**

Several studies have reported the use of acellular dermal matrix (ADM) for positioning the IMF mainly in breast augmentation patients.  It provides inferior and lateral IMF support, stabilising an implant’s position. It also has benefits in revisionary surgery particularly for capsular contracture and implant malposition.

Safran^74^ challenged existing views on the use of ADM in pre-pectoral implant-based post-mastectomy breast reconstruction using a Wise-pattern wide-based dermal-subcutaneous flap for IMF reconstruction. The ADM was wrapped around the anterior surface of the implant, it was used in this manner to reduce capsular contracture of the implant and to provide an additional layer to the reconstructive flap, reducing skin flap compliance and risk of bottoming-out. However, this study did not show statistically significant difference in rates of capsular contracture, surgical or aesthetic complications when ADM was not used.

There is a deficit of studies reporting on ADM use in revisionary breast reconstruction as opposed to primary augmentation and reconstruction^45^. Safran adds to the literature on ADM use in primary reconstruction. High success rates for correcting IMF malposition using ADM have been reported compared to other revisionary reconstruction techniques^75^. The benefit of ADM to correct other implant related deformities including capsular contraction, ptosis and implant visibility have also been reported^30,76-79^. Further studies into ADM use in tuberous breast and male-to-female augmentation where these complications are common would be valuable.

**Additional References**

73. Ospital C., Delay E., Grolleau J.L., Henry G., Mojallal A.A. Primary management of the inframammary fold in breast reconstruction using the thoracoabdominal advancement flap: surgical technique. Ann Chir Plast Esthet. 2024;69(2):178–185.

74. Safran T., Al-Halabi B., Viezel-Mathieu A., Boileau J.F., Dionisopoulos T. Direct-to-implant, prepectoral breast reconstruction: a single-surgeon experience with 201 consecutive patients. Plast Reconstr Surg. 2020;145(4):686e–696e.

75. Spear S.L., Sher S.R., Al-Attar A., Pittman T. Applications of acellular dermal matrix in revision breast reconstruction surgery. Plast Reconstr Surg. 2014;133(1):1–10.

76. Baxter R.A. Intracapsular allogenic dermal grafts for breast implant-related problems. Plast Reconstr Surg. 2003;112(6):1692–1696. discussion 7-8.

77. Breuing K.H., Colwell A.S. Inferolateral AlloDerm hammock for implant coverage in breast reconstruction. Ann Plast Surg. 2007;59(3):250–255.

78. Grabov-Nardini G., Haik J., Regev E., Winkler E. AlloDerm sling for correction of synmastia after immediate, tissue expander, breast reconstruction in thin women. Eplasty. 2009;9:e54.

79. Hartzell T.L., Taghinia A.H., Chang J., Lin S.J., Slavin S.A. The use of human acellular dermal matrix for the correction of secondary deformities after breast augmentation: results and costs. Plast Reconstr Surg. 2010;126(5):1711–1720.
